# Fasting plasma glucose is an independent predictor of survival in patients with locally advanced non-small cell lung cancer treated with concurrent chemoradiotherapy

**DOI:** 10.1186/s12885-019-5370-5

**Published:** 2019-02-21

**Authors:** Milana Bergamino, Antonio J. Rullan, Maria Saigí, Inmaculada Peiró, Eduard Montanya, Ramón Palmero, José Carlos Ruffinelli, Arturo Navarro, María Dolores Arnaiz, Isabel Brao, Samantha Aso, Susana Padrones, Felipe Cardenal, Ernest Nadal

**Affiliations:** 1 Department of Medical Oncology, Thoracic Oncology Division, Catalan Institute of Oncology, Hospital Duran i Reynals, Avda Gran via, 199-203, L’Hospitalet, 08908 Barcelona, Spain; 2 Clinical Nutrition Unit, Catalan Institute of Oncology, Hospital Duran i Reynals, L’Hospitalet, Barcelona, Spain; 3 Department of Endocrinology, Hospital Universitari de Bellvitge, L’Hospitalet, Barcelona, Spain; 4 Department of Clinical Sciences, University of Barcelona, Hospital Universitari de Bellvitge, IDIBELL, CIBERDEM, L’Hospitalet, Barcelona, Spain; 5 Department of Radiation Oncology, Catalan Institute of Oncology, Hospital Duran i Reynals, L’Hospitalet, Barcelona, Spain; 6 Department of Respiratory Medicine, Hospital Universitari de Bellvitge, L’Hospitalet, Barcelona, Spain; 7 Clinical Research in Solid Tumors Group (CReST), Oncobell Program, Bellvitge Biomedical Research Institute (IDIBELL), L’Hospitalet, Barcelona, Spain

**Keywords:** Locally advanced unresectable non-small cell lung cancer, Concurrent chemoradiotherapy, Hyperglycemia, Type 2 diabetes mellitus, Comorbidities

## Abstract

**Background:**

Diabetes is related with increased cancer mortality across multiple cancer types. Its role in lung cancer mortality is still unclear. We aim to determine the prognostic value of fasting plasma glucose (FPG) and diabetes mellitus in patients with locally advanced non-small cell lung cancer (NSCLC) treated with concurrent chemoradiotherapy.

**Methods:**

One-hundred seventy patients with stage III NSCLC received definitive concurrent chemoradiotherapy from 2010 to 2014. Clinico-pathological data and clinical outcome was retrospectively registered. Fifty-six patients (33%), met criteria for type 2 diabetes mellitus (T2DM) at baseline. The prognostic value of FPG and other clinical variables was assessed. Overall survival (OS) and progression-free survival (PFS) were estimated using the Kaplan–Meier method and Cox proportional models and log-rank test were used.

**Results:**

With a median follow-up of 36 months, median PFS was 8.0 months and median OS was 15.0 months in patients with FPG ≥7 mmol/L compared to 20 months (HR 1.13; 95% CI 1.07–1.19, *p* < 0.001) and 31 months (HR 1.09; 95% CI 1.04–1.15; p < 0.001) respectively, for patients with FPG < 7 mmol/L. In the multivariate analysis of the entire cohort adjusted by platinum compound and comorbidities, high levels of FPG as a continuous variable (HR 1.14; 95% CI 1.07–1.21; p < 0.001), the presence of comorbidity (HR 1.72; 95% CI 1.12–2.63; *p* = 0.012), and treatment with carboplatin (HR 1.95; 95% CI 1.26–2.99; *p* = 0.002) were independent predictors for shorter OS. In additional multivariate models considering non-diabetic patients as a reference group, diabetic patients with poor metabolic control (HbA1c > 8.5%) (HR 4.53; 95% CI 2.21–9.30; p < 0.001) and those receiving insulin (HR 3.22; 95% CI 1.90–5.46 p < 0.001) had significantly independent worse OS.

**Conclusion:**

Baseline FPG level is an independent predictor of survival in our cohort of patients with locally advanced NSCLC treated with concurrent chemoradiotherapy. Studies in larger cohorts of patients are warranted to confirm this relevant association.

**Electronic supplementary material:**

The online version of this article (10.1186/s12885-019-5370-5) contains supplementary material, which is available to authorized users.

## Key message

In this study, we determine the prognostic value of fasting plasma glucose (FPG) and Type 2 Diabetes Mellitus (T2DM) in patients with stage III non-small cell lung cancer treated with concurrent chemoradiotherapy. High FPG at baseline predicts worse clinical outcome independently of other clinical variables. Metabolic control and antidiabetic treatment might also influence outcome in diabetic patients.

## Background

Diabetes has been associated with an increased incidence and mortality in many types of cancer [1–3]. Diabetes may influence cancer progression and outcome by several mechanisms, including hyperinsulinemia, hyperglycemia, or chronic inflammation. In addition, type 2 diabetes mellitus (T2DM) is a risk factor for other non-neoplastic causes of death in cancer patients [4].

The role of diabetes in the prognosis of patients with lung cancer has not been well established [5]. Some clinical and epidemiologic studies showed that pre-existing diabetes has a negative impact on lung cancer mortality [5–7]. A worse prognosis in diabetic patients with lung cancer has been mainly seen in women [6], patients with non-small cell lung cancer (NSCLC) and in those patients treated with surgical resection [5]. However, these observational studies have methodological limitations such as the absence of screening for T2DM [2], the lack of cause-specific death reporting and the heterogeneity of tumor stage at cancer diagnosis [7].

The use of baseline glycemia as a continuous variable could avoid the problem of T2DM underdiagnosis. In fact, fasting plasma glucose (FPG) level has been independently associated with an increased risk of mortality in a cohort of patients with newly diagnosed NSCLC [7]. In addition, The Emerging Risk Factors Collaboration Study analyzed the potential independent associations between diabetes or hyperglycemia with the risk of death from cancer. This study showed an association between high FPG levels (exceeding 100 mg per deciliter or 5.6 mmol per liter)and cancer specific mortality [8].

The influence of antidiabetic therapy on cancer incidence and mortality has been the subject of many observational epidemiologic studies in large populations [9, 10]. These studies suggest that metformin is associated with a decrease in cancer mortality, while data regarding other antidiabetic medications are conflicting [10, 11]. The Danish Cancer Registry provided evidence that among cancer patients with pre-existing diabetes, mortality rates were higher on patients receiving insulin [12].

The purpose of our study is to determine the influence of T2DM and FPG levels on survival in a cohort of patients with unresectable locally advanced NSCLC treated with concurrent chemoradiotherapy. The influence on overall survival (OS) of antidiabetic treatments and glycemic control among T2DM patients were also evaluated as secondary endpoints.

## Methods

### Study population

Eligible patients for this study had histological or cytological confirmation of NSCLC, locally advanced disease based on clinical assessments: cardiopulmonary function, contrast thoracic computed tomography (CT) scan and positron-emitted tomography-CT (PET-CT) scan, magnetic resonance imaging (MRI) of the brain and selective mediastinal staging with endobronquial ultrasonography and/or endoscopic (EBUS/END) ultrasonography; had been considered candidates for non-surgical cancer treatment by the Multidisciplinary Thoracic Oncology Board and had received definitive concurrent chemoradiation. From January 2010 to December 2014 the medical records of 170 consecutive patients from our institution fulfilling all those criteria were reviewed. We collected the following data: age, sex, smoking history, weight loss during the 6 months previous to NSCLC diagnosis, Eastern Cooperative Oncology Group performance status (ECOG PS), histology and clinical stage according to the 7th edition of TNM classification [13], chemotherapy regimen, total dose of radiotherapy and treatment-related toxicity. We did not use any established comorbidity scale such as Charlson Comorbidity Index or the Simplified Comorbidity Score due to the strong influence of T2DM on those scales [14]. Presence of relevant comorbidities was registered (renal insufficiency, chronic obstructive pulmonary disease (COPD) cardiovascular disease and chronic hepatic disease). Thoracic radiotherapy was administered up to a total dose of 60 to70 Gy (1.8–2 Gy per fraction over 6–7 weeks of treatment). Chemotherapy regimens, starting the first day of radiotherapy, were based on cisplatin or carboplatin combined with etoposide, vinorelbine, pemetrexed or paclitaxel. None of the patients received consolidation with durvalumab after completing chemoradiotherapy. Patients were followed-up until June 2017. This study was approved by the Institutional Review Board and recorded data were anonymized for analysis.

### Diabetes evaluation and treatment

Patients had been assessed before cancer treatment by a nutritionist plus/minus an endocrinologist both working as a team at our Institution. Patients were classified as having T2DM if the diagnosis was in their medical record, if they were on antidiabetic drugs or if they met the diagnostic criteria for diabetes according to the American Diabetes Association (ADA) Criteria from 2016 [15]. FPG measured less than 1 week before starting cancer treatment was considered the baseline glycemia. Hyperglycemia was defined as FPG ≥ 7 mmol/L (≥126 mg/dL). Last available glycated hemoglobin (HbA1c) was recorded for diabetic patients with a maximum of 6 months before starting oncologic treatment. T2DM patients were classified into three groups of glycemic control: good (HbA1c ≤ 7%), moderate (HbA1c 7.1–8.5%) and poor (HbA1c > 8.5%) [9]. Those antidiabetic drugs that the patient was receiving when oncologic treatment was started were collected.

### Statistical analysis

Chi square test for categorical and T- Student test for continuous variables were used to compare the characteristics of T2DM and non-diabetic patients. Overall survival (OS) was defined as the time between lung cancer diagnosis and death, progression free survival (PFS) was defined as the time between histological diagnosis and radiological progression of the disease or death, whichever came first. Patients with no evaluable radiological images were censored. Kaplan–Meier method was used to estimate OS and PFS. The Cox proportional hazard regression model and the log-rank test were used to assess differences among prognostic factors. Multivariate survival analysis was performed using Cox Regression with the Forward Step model. A two-tailed *p-*value of less than 0.05 was considered statistically significant. All analyses were carried out using the SPSS 22.0 statistical software (SPSS Inc., Chicago, IL, USA).

## Results

### Patient characteristics

In this cohort of 170 patients, 56 (33%) met T2DM criteria. There were no patients with type 1 diabetes mellitus. Baseline characteristics, including gender, smoking history, ECOG PS, comorbidity and stage were similar in patients with T2DM and in non-diabetic patients (Table 1). Diabetic patients were more likely to have squamous tumors (51.8% vs 37.7%, *p* = 0.004) and to receive carboplatin regimens (51.7%) compared with non-diabetic patients (32.5%) (*p* = 0.019). Median number of cycles of chemotherapy and the total dose of radiation therapy received were similar in T2DM and nondiabetic patients. There were no statistically significant differences in comorbidities between T2DM and non-diabetic patients, with the exception of cardiovascular disease that was more common in diabetic patients (33.3% vs 13.8%, *p* = 0.003).

**Table 1:** Baseline characteristics

	T2DM (*n* = 56)	Non T2DM (*n* = 114)	All(*n* = 170)	*p*-value
Age,median (range)	66 (49–81)	63 (37–87)	64 (37–87)	0.094
Gender,n (%)				
Male	51 (91.1%)	97 (85.1%)	148 (87%)	
Female	5 (8.9%)	17 (14.9%)	22 (13%)	0.337
Smoking history, n (%)				
Current	27 (48.2%)	57 (50%)	84 (49.4%)	
Former	24 (42.8%)	51 (44.7%)	75 (44.1%)	
Never	3 (5.4%)	4 (3.5%)	7 (4.1%)	
Unknown	2 (3.6%)	2 (1.8%)	4 (2.4%)	0.837
ECOG PS, n (%)				
PS 0–1	53 (94.6%)	102 (90.3%)	155 (91.1%)	
PS 2	3 (5.4%)	12 (9.7%)	15 (8.9%)	0.331
Histology n (%)				
Adenocarcinoma	9 (16%)	48 (42.1%)	57 (33.5%)	
Squamous	29 (51.8%)	43 (37.7%)	72 (42.3%)	
NOS	18 (32.2%)	23 (20.2%)	41 (24.2%)	0.003
Stage, n (%)				
IIIA	31 (55.3%)	54 (47.4%)	85 (50%)	
IIIB	23 (41.1%)	58 (50.9%)	81 (47.6%)	0.321
Mean baseline glycemia (mmol/L)	9.22 ± 6.35	5.6 ± 1.2	6.75 ± 4.70	< 0.001
Comorbidities				
Any	34 (60.7%)	69 (60.5%)	103 (60.6%)	0.981
Renal Insufficiency	5 (8.9%)	7 (6.1%)	12 (7.1%)	0.445
COPD	21 (37.5%)	50 (44%)	71 (41.8%)	0.604
Cardiovascular Hepatopathy	18 (32.1%) 2 (3.6%)	16 (14%) 0 (0%)	34 (20%) 2 (1.2%)	0.003 1.000
Platinum doublet, n (%)				
Cisplatin	27 (48.2%)	77 (67.5%)	104 (61.2%)	
Carboplatin	29 (51.7%)	37 (32.5%)	66 (38.8%)	0.019
Total dose of RDT between 60-70Gys	54 (96.4%)	106 (93%)	160 (94%)	0.716

Abbreviations: *ECOG PS* Eastern Cooperative Oncology Group performance status, *COPD* chronic obstructive pulmonary disease, *NOS* not otherwise specified, *RDT* radiotherapy, *T2DM* type 2 diabetes mellitus

Among T2DM patients, 39 (69.6%) were receiving antidiabetic medication at lung cancer diagnosis. Twenty patients were receiving metformin alone or in combination with other oral hypoglycemic agents and 19 were on insulin treatment alone or with oral hypoglycemic drugs. Relevant baseline nutritional characteristics are shown in Additional file 1: Table S1. Overweight and obesity were more likely in T2DM patients. No statistically significant differences were observed in terms of type of chemotherapy received concurrently with thoracic radiotherapy as shown in Additional file 1: Table S2.

### Overall survival

After a median follow-up of 36 months, 47 patients (27.6%) were alive. Median PFS was 12 months (95% CI 9–15) and median OS was 26 months (95% CI 20–32) for the whole cohort. Major causes of death were tumor progression (80%), treatment-related adverse events (7.8%), cardiovascular disease (7%), other causes (3.5%) and second tumors (1.7%). There were no significant differences in causes of death between diabetic and non-diabetic patients (*p* = 0.402). There were no significant differences in the pattern of tumor progression among diabetic and non-diabetic patients (*p* = 0.274).

### Univariate survival analysis according to T2DM related variables

**Fig. 1:**
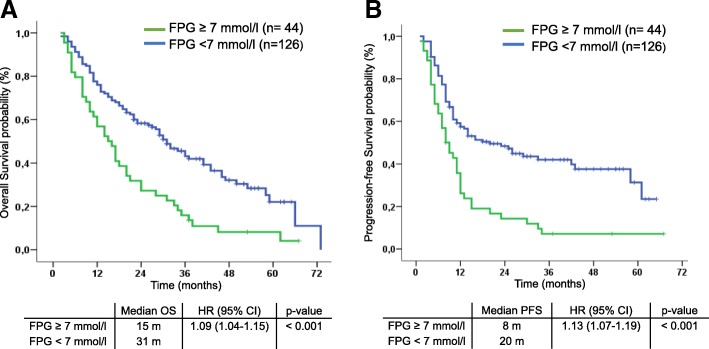
Kaplan Meier curves for overall survival (**a**) and progression-free survival (**b**) according to pre-treatment Fasting plasma glucose (FPG) in the whole cohort (*n* = 170). Patients with FPG ≥7 mmol/L had significantly shorter median OS and PFS compared with patients with FPG < 7 mmol/L. *Abbreviations: Overall Survival (OS), Progression Free Survival (PFS)*

Median OS was significantly shorter in patients with FPG ≥ 7 mmol/L (15 months) compared to patients with FPG < 7 mmol/L (31 months, HR 1.09; 95% CI 1.04–1.15; *p* < 0.001; Fig. 1a). Median PFS was also significantly shorter in patients with high FPG compared to patients with FPG < 7 mmol/L (8 vs 20 months; HR 1.13; 95% CI 1.07–1.19; p < 0.001; Fig. 1b).

**Fig. 2:**
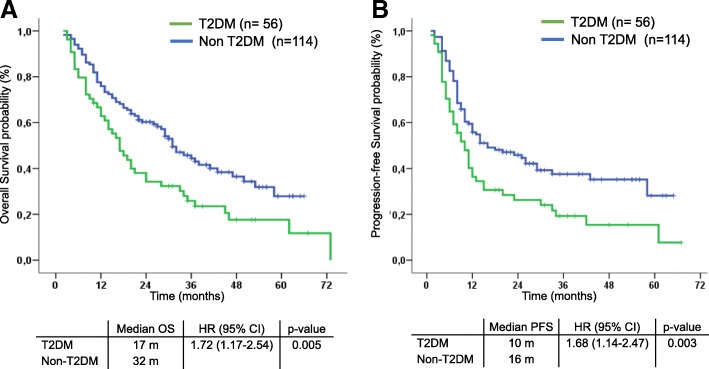
Kaplan Meier curves for overall survival (**a**) and progression-free survival (**b**) according to diagnosis of type 2 diabetes mellitus (T2DM) in the whole cohort (*n* = 170). Patients with T2DM diagnosis had significantly shorter median OS and PFS compared with patients without T2DM history. *Abbreviations: Overall Survival (OS), Progression Free Survival (PFS)*

Median OS was significantly inferior in T2DM patients (17 months) compared to non-diabetic patients (32 months, HR 1.72; 95% CI 1.17–2.54; *p* = 0.005; Fig. 2a). Median PFS was significantly inferior in T2DM as well (10 vs 16 months; HR 1.68; 95% CI 1.14–2.47; *p* = 0.003; Fig. 2b).

**Fig. 3:**
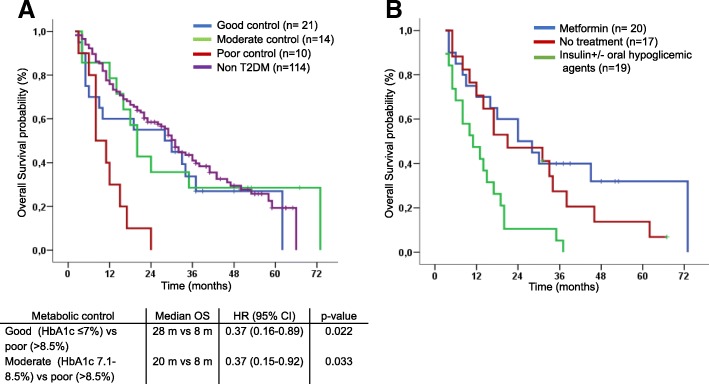
Kaplan Meier curves for OS in type 2 T2DM and non-diabetic patients according to metabolic control based on HbA1 (**a**) and type of anti-diabetic treatment (**b**). Patients with poor metabolic control (HbA1c > 8.5%) had shorter median OS as compared with the rest of diabetic patients and nondiabetic patients. Patient receiving insulin had also worse OS compared with the rest of diabetic patients. *Abbreviations: Type 2 Diabetes Mellitus (T2DM), Overall survival (OS), Glycated Haemoglobin (HbA1c)*

HbA1c levels were available in 45 patients with T2DM. Significant differences in median OS were observed between patients with good (28 months; *n* = 21) and moderate metabolic control (20 months; *n* = 14) compared to those with poor metabolic control (8 months; *n* = 10) (HR 0.37; 95% CI 0.16–0.89, *p* = 0.022; HR 0.37; 95% CI 0.15–0.92; *p* = 0.033, respectively; Fig. 3a). No statistically significant differences were found in OS between T2DM patients with good metabolic control and non-diabetic patients (28 vs 31 months; *p* = 0.34). Interestingly, differences in OS were observed according to antidiabetic treatment. Patients treated with insulin had significantly shorter median OS compared to those receiving metformin or not treated with antidiabetic agents (11 m vs 24 m vs 21 m, respectively; *p* = 0.001; Fig. 3b).

Patients with poor metabolic control (HbA1c > 8.5%) did not have significantly higher presence of comorbidity (Additional file 1: Table S3). Patients receiving insulin were more likely to have poor metabolic control (*p* = 0.045, Additional file 1: Table S4).

### Multivariate analysis

We performed a multivariate Cox regression analysis including all patients in the study. We introduced prognostic factors previously described in NSCLC patients (age, histology, smoking status, ECOG PS, platinum treatment, comorbidity, BMI, weight loss before treatment, nodal status). Pre-treatment FPG level (HR 1.13; 95% CI 1.06–1.21; *p* < 0.001) was an independent prognostic factor, whereas T2DM diagnosis was not (Additional file 1: Table S5). The type of platinum treatment (HR 1.66; 95%CI 1.12–2.45; *p* = 0.011) and the presence of comorbidity (HR 1.51; 95% CI 1.02–2.23; *p* = 0.039) were independent prognostic factors for OS as well. However, weight loss was not associated with overall survival in the multivariate analysis.

We constructed two additional models for metabolic control of diabetes and insulin treatment, with non-diabetic patients as the reference group. In the multivariate analysis, poor metabolic control (HbA1c > 8.5%) (HR 4.53; 95% CI 2.21–9.30; p < 0.001) and insulin treatment (HR 3.22; 95% CI 1.91–5.46; p < 0.001) were independent prognostic factors when adjusted for the above-mentioned variables (Table 2).

**Table 2:** Multivariate analysis for Overall Survival for selected prognostic factors in NSCLC. A model was built for each variable of interest. All the variables listed at the upper file were included in the multivariabte Cox model, but the hazard ratios are shown only for those covariates that remained statistically significant during the forward stepwise analysis

Model 1					
Covariates	Age, Histology, Smoking status, ECOG PS, platinum treatment, comorbidity, BMI, weight loss, nodal status, pre-treatment fasting plasma glucose (FPG).				
Variables in the equation	Parameter	Level	Hazard Ratio	95% Confidence Interval	*p*-value
	Pre-treatment FPG	Continuous	1.134	1.066–1.207	< 0.001
	Platinum treatment	Carboplatin vs cisplatin	1.657	1.121–2.451	0.011
	Comorbidity	Yes vs No	1.508	1.020–2.229	0.039
Model 2					
Covariates	Age, Histology, Smoking status, ECOG PS, platinum treatment, comorbidity, BMI, weight loss, nodal status, metabolic control groups.				
Variables in the equation	Parameter	Level	Hazard Ratio	95% Confidence Interval	*p*-value
	Metabolic control groups	No diabetes (reference group)			
		HbA1c ≤7	1.342	0.752–2.395	0.319
		HbA1c 7.1–8.5	0.830	0.400–1.719	0.616
		HbA1c > 8.5	4.534	2.210–9.301	< 0.001
	Platinum treatment	Carboplatin vs cisplatin	1.946	1.264–2.996	0.002
	Comorbidity	Yes vs No	1.720	1.125–2.632	0.012
Model 3					
Covariates	Age, Histology, Smoking status, ECOG PS, platinum treatment, comorbidity, BMI, weight loss, nodal status, insulin treatment.				
Variables in the equation	Parameter	Level	Hazard Ratio	95% Confidence Interval	*p*-value
	Insulin treatment	Yes vs No	3.225	1.906–5.456	< 0.001
	Platinum treatment	Carboplatin vs cisplatin	1.574	1.075–2.306	0.020

Abbreviations: *BMI* body mass index, *ECOG PS* Eastern Cooperative Oncology Group performance status, *FPG* fasting plasma glucose, *HbA1c* glycated haemoglobin

## Discussion

In this homogeneous cohort of consecutive patients with unresectable locally advanced NSCLC, both pre-treatment FPG level and diagnosis of T2DM were predictors of overall survival. However, only FPG level retained its significance in the multivariate analysis. Interestingly, PFS, a cancer-related outcome, was significantly shorter for patients with high FPG, suggesting a direct relationship between both variables.

Several mechanisms may explain the negative effect of hyperglycemia and diabetes on lung cancer related outcome. Hyperinsulinemia and metabolic rewiring of cancer cells are potential factors contributing to the development and progression of cancer. According to Warburg’s hypothesis, the hyperglycemic environment may accelerate the proliferation of cancer cells, as they can obtain essential metabolites and energy mainly from fermentation of glucose, even in aerobic conditions [16, 17]. Data from patients and mouse models indicates that lung tumors are dependent on glucose metabolism, and increased expression of glycolytic enzymes correlate with poor prognosis. In our study, squamous cell carcinoma histology was more probable in diabetic patients. A recent case-control study showed an increased risk of lung cancer associated with glycemic load and dietary glycemic index, as a marker of carbohydrate intake and postprandial glucose response. The risk was greater for the development of squamous cell carcinoma [18]. In another study, squamous cell lung carcinoma was associated with enhanced glucose uptake and exhibited higher glycolytic dependency than adenocarcinoma [19].

Han et al. found that high glucose levels can promote cancer proliferation via the induction of epidermal growth factor (EGF) expression and transactivation of EGF receptor [20]. Hyperglycemia has also shown to decrease the antiproliferative effect of chemotherapy in preclinical models, but the results are inconsistent and have not been proven in clinical trials [21]. Some retrospective series have also shown a negative impact on survival of elevated blood glucose levels during radiation therapy in glioblastomas. This detrimental effect may be explained by the metabolic impact on tumor microenvironment, but also by the induction of hypoxia that promotes resistance to radiotherapy [22, 23].

The association of high FPG with outcome in lung cancer patients in a clinical setting has already been reported. In an unselected cohort of 342 patients with newly diagnosed NSCLC, Luo et al. observed that patients with FPG levels ≥7.0 mmol/L (≥ 126 mg/dl) had shorter survival outcome independently of other prognostic factors [7]. In another retrospective study, 159 patients with locally advanced NSCLC were treated with radical chemoradiotherapy and those patients who had a previous diagnosis of diabetes or with FPG ≥ 7.0 mmol/L had a significantly shorter survival independently of other prognostic factors [24]. In this study, FPG level was not studied in a separate model in the multivariate analysis, and the value of this parameter at baseline as a continuous variable was not clarified. By contrast, in our study, previous diagnosis of T2DM lost its statistical significance in the multivariate analysis, whereas FPG maintained a strong effect.

To evaluate the influence of metabolic control of diabetes on cancer outcome, we explored the association of HbA1c (as a dichotomous variable) with the prognosis of lung cancer. We observed that diabetic patients with the poorest glycemic control (HbA1c > 8.5%) had the shortest survival, and that diabetic patients with good metabolic control (HbA1c < 7%) had a similar OS than non-diabetic patients. Moreover in a multivariate model poor metabolic control was independently related to prognosis. These findings underline the potential benefit of achieving good metabolic control to improve cancer outcome in diabetic patients [25].

The influence of antidiabetic treatment on cancer prognosis is still controversial. Metformin is an antidiabetic drug that suppresses hepatic neoglucogenesis and improves insulin sensitivity. Preclinical data have shown that metformin has direct and indirect potent antitumor effects in several cancers, including lung. However the concentration of metformin used in these studies is about one thousand times higher than that achieved in the blood in humans [26]. Some epidemiological studies in lung cancer have shown better outcomes in metformin-treated patients compared to diabetic patients receiving other antidiabetic drugs [10, 11, 27]. Ahmed I et al., in a study with 166 patients, did not find any differences in survival between diabetic and non-diabetic patients except for being on metformin treatment or not [28]. More than 200 clinical trials [10] are ongoing aiming to determine whether the preclinical anticancer effect of metformin translates into clinical benefit. The Danish Cancer Registry showed a higher mortality among patients treated with insulin [12]. In line with those reports, we observed that patients treated with insulin (± oral hypoglycemic drugs) had a worse prognosis than patients on metformin treatment (without insulin) or those that did not receive any antidiabetic treatment.

A common concern when studying the relationship between diabetes and cancer prognosis is the higher prevalence of comorbidities in diabetic patients and the existence of competing causes of death. We did not identify significant differences in the cause of death between diabetic and non-diabetic patients. Although patients with T2DM had higher prevalence of overweight, body mass index (BMI) was not a significant prognostic factor in our study. We also collected basic malnutrition measures as weight loss before treatment and baseline albumin level as known prognostic factors. However, none of these factors was different between the T2DM and the non-diabetic patients or achieved significance in the survival analysis. Another prominent issue is that the presence of T2DM could lead to a less intensive anticancer treatment. Although diabetic patients were more likely treated with carboplatin than cisplatin and, carboplatin treatment was associated with unfavorable overall survival in the multivariate analysis, baseline FPG, metabolic control and insulin treatment remained independent prognostic factors after adjusting by platinum treatment.

The major limitation of our study is the relatively low sample size, which could lead to residual confounding as we do not have enough power to identify some well-known risk factors and compromises the analysis of subsets of patients. Another weakness is the retrospective nature of the study, which means that there was not a dedicated pre-planned design of patient management and data collection. The strength of our study is the uniform clinical practice on cancer and diabetic management at a single institution. The most relevant finding is that baseline FPG level is a strong predictor of survival in a set of consecutive patients treated with definitive chemoradiotherapy. Our results on the prognostic value of metabolic control and antidiabetic treatment have potential clinical implications and are consistent with what has been reported in the literature, but should be regarded with caution due to the limitations of the study.

## Conclusions

Our data support that baseline FPG level may impact on the outcome of patients with locally advanced NSCLC. The validation of this association in a large cohort of locally advanced NSCLC patients is warranted. In the complex relationship between cancer and diabetes our results suggest that metabolic control and treatment of diabetes might influence cancer-related outcomes. Prospective research in the field of diabetic control and management in lung cancer patients is necessary to provide insight into these issues.

## Additional file

Additional file 1: **Table S1**. Nutritional baseline characteristics in T2DM and non-diabetic patients. **Table S2.**Chemotherapy regimens given concurrently with thoracic radiotherapy in T2DM and non-diabetic patients. **Table S3.**Presence of comorbidity according to metabolic control in T2DM patients (*n* = 45). **Table S4.**Metabolic control based on HbA1c according to antidiabetic treatment (*n* = 45). **Table S5**.Multivariate analysis for Overall Survival for selected prognostic factors in NSCLC including T2DM as covariate. (DOCX 20 kb)

## Abbreviations

ADA: American Diabetes Association
BMI: Body Mass Index
COPD: Chronic obstructive pulmonary disease
CT: Computed tomography
EBUS/EUS: Endobronchial/endoscopic ultrasound
ECOG PS: Eastern Cooperative Oncology Group performance status
FPG: Fasting plasma glucose
HbA1c: Glycated hemoglobin
NSCLC: Locally advanced non-small cell lung cancer
OS: Overall survival
PET-CT: Positron emission tomography-CT
PFS: Progression-free survival
T2DM: Type 2 Diabetes Mellitus
