# Supplementary material for: Fasting plasma glucose is an independent predictor of survival in patients with locally advanced non-small cell lung cancer treated with concurrent chemoradiotherapy
Source: BMC Cancer. 2019 Feb 21;19:165. doi: 10.1186/s12885-019-5370-5 (PMC6385407; doi:10.1186/s12885-019-5370-5)
Supplement: Supplementary file 1 — Table S1. Nutritional baseline characteristics in T2DM and non-diabetic patients. Table S2.Chemotherapy regimens given concurrently with thoracic radiotherapy in T2DM and non-diabetic patients. Table S3.Presence of comorbidity according to metabolic control in T2DM patients (n = 45). Table S4.Metabolic control based on HbA1c according to antidiabetic treatment (n = 45). Table S5.Multivariate analysis for Overall Survival for selected prognostic factors in NSCLC including T2DM as covariate. (DOCX 20 kb) [file 12885_2019_5370_MOESM1_ESM.docx]

**Additional file 2**

**Table S1.**Nutritional baseline characteristics in T2DM and non-diabetic patients.

|  | **T2DM (n=56)** | **Non T2DM (n=114)** | **General (n=170)** | **p-value** |
| --- | --- | --- | --- | --- |
| **BMI, n (%)**  Underweight (<18)  Normal (18-24.9)  Overweight (25-29.9)  Obesity (>30) | 0 (0%)  10 (17.9%)  28 (50%)  18 (22.1%) | 2 (1.8%)  44 (38.6%)  45 (39.4%)  23 (20.2%) | 2 (1.2%)  54 (31.8%)  72 (42.4%)  42 (24.6%) | **0.03** |
| **Weight loss before treatment, n (%)**  <5%  5-10%  >10% | 43 (76.8%)  9 (16.1%)  4 (7.1%) | 87 (76.3%)  21 (18.4%)  6 (5.2%) | 130 (66.5%)  30 (17.6%)  10 (5.9%) | 0.757 |
| **Mean baseline albumin (g/L)** | 40.5 (24-51) | 42 (29-50) | 41.5 (24-51) | 0.323 |
| **Albumin decrease during treatment, n (%)**  **<10%**  **>10%** | 42 (75%)  14 (25%) | 76 (66.6%)  38 (33.3%) | 118 (68%)  52 (32%) | 0.217 |

**Abbreviations:** BMI, body mass index; T2DM, type 2 diabetes mellitus.

**Table S2.**Chemotherapy regimens given concurrently with thoracic radiotherapy in T2DM and non-diabetic patients.

|  | **T2DM (n=56)** | **Non T2DM (n=114)** | **All (n=170)** | **p-value** |
| --- | --- | --- | --- | --- |
| **Cycles given concurrently**  (median (range)) | 2 (1-3) | 2 (1-3) | 2 (1-3) | 0.146 |
| **Chemotherapy regimen**  CDDP-VP16 | 26 (48.1%) | 69 (59.5%) | 95 (55.9%) | 0.172 |
| CDBCA-VP16 | 11 (20.4%) | 18 (15.5%) | 29 (17.1%) |  |
| CDBCA-Taxol | 4 (7.4%) | 2 (1.7%) | 6 (3.5%) |  |
| CDBCA-VNR | 10 (18.5%) | 18 (15.5%) | 28 (16.5%) |  |
| CDDP-VNR | 0 (0%) | 5 (4.3%) | 5 (2.9%) |  |
| CDDP-Pemetrexed | 1 (1.9%) | 3 (2.6%) | 4 (2.4%) |  |
| CDBCA | 2 (3.7%) | 1 (0.9%) | 3 (1.8%) |  |
| **Consolidation CT**  Yes | 10 (18.5%) | 35 (30.2%) | 45 (26.5%) | 0.136 |

**Abbreviations**: CDDP, cisplatin; CBDCA, carboplatin; CT, chemotherapy; VNR vinorelbine; VP16, etoposide.

**Table S3.**Presence of comorbidity according to metabolic control in T2DM patients (n=45).

| **Metabolic control** | **No Comorbidity**  **(n=17)** | **Any comorbidity**  **(n=28)** | **All**  **(n=45)** | **p-value** |
| --- | --- | --- | --- | --- |
| HbA1c ≤ 7% | 9 (43%) | 12 (57%) | 21 (45%) | 0.774 |
| HbA1c 7.1-8.5% | 5 (35%) | 9 (65%) | 14 (32%) |  |
| HbA1c >8.5% | 3 (30%) | 7 (70%) | 10 (23%) |  |

**Abbreviations:** HbA1c, glycated haemoglobin, T2DM, type 2 diabetes mellitus.

*From the fifty-six (33%) patients who met T2DM criteria, HbA1c levels were not available in 11 patients.

**Table S4.**Metabolic control based on HbA1c according to antidiabetic treatment (n=45).

| **Metabolic control** | **No Insulin**  **(n=28)** | **Insulin**  **(n=17)** | **All**  **(n=45)** | **p-value** |
| --- | --- | --- | --- | --- |
| HbA1c ≤ 7% | 16 (57%) | 5 (29.5%) | 21 (45%) | 0.045 |
| HbA1c 7.1-8.5% | 9 (32%) | 5 (29.5%) | 14 (32%) |  |
| HbA1c >8.5% | 3 (11%) | 7 (41%) | 10 (23%) |  |

**Abbreviations**: HbA1c, glycated haemoglobin; T2DM, type 2 diabetes mellitus.

*From the fifty-six (33%) patients who met T2DM criteria, HbA1c levels were not available in 11 patients.

**Table S5**.Multivariate analysis for Overall Survival for selected prognostic factors in NSCLC including T2DM as covariate.

| **Parameters** | | **Hazard Ratio** | **95% Hazard Ratio Confidence Intervals** | | **p-value** |
| --- | --- | --- | --- | --- | --- |
| **Age** | Continuous | 0.991 | 0.966 | 1.017 | 0.513 |
| **Histology** | Adenocarcinoma vs Squamous vs Others |  |  |  | 0.062 |
|  | Adenocarcinoma vs Others | 0.574 | 0.332 | 0.995 | **0.048** |
|  | Squamous vs Others | 1.023 | 0.629 | 1.663 | 0.927 |
| **Smoking history** | Current vs former vs never |  |  |  | 0.730 |
|  | Current vs never | 0.703 | 0.249 | 1.663 | 0.507 |
|  | Former vs never | 0.664 | 0.238 | 1.853 | 0.434 |
| **ECOG PS** | PS 0-1 vs PS2 | 1.507 | 0.708 | 3.210 | 0.287 |
| **N status** | N0-1 vs N2-3 | 0.603 | 0.384 | 1.044 | 0.071 |
| **Weight loss before treatment** | Continuous | 1.026 | 0.975 | 1.079 | 0.318 |
| **BMI before treatment** | Continuous | 1.007 | 0.958 | 1.057 | 0.793 |
| **Comorbidity** | Yes vs No | 1.465 | 0.937 | 2.290 | 0.094 |
| **Platinum treatment** | CDBCA vs CDDP | 2.170 | 1.317 | 3.575 | **0.002** |
| **T2DM** | Yes vs No | 0.876 | 0.574 | 1.338 | 0.540 |

**Abbreviations:** BMI, body mass index; ECOG PS, Eastern Cooperative Oncology Group performance status; N status: nodal status; T2DM, type 2 diabetes mellitus
